# Zebrafish mutants of the neuromuscular junction: swimming in the gene pool

**DOI:** 10.1007/s12576-015-0372-9

**Published:** 2015-03-18

**Authors:** Eriko Daikoku, Masahisa Saito, Fumihito Ono

**Affiliations:** 1 Department of Physiology, Osaka Medical College, Takatsuki, 569-8686 Japan; 2 Laboratory of Molecular Physiology, NIAAA, NIH, Bethesda, MD 20892 USA

**Keywords:** Synapse, Zebrafish, Acetylcholine receptor, Mutant

## Abstract

This review provides an overview of zebrafish mutants with dysfunctional acetylcholine receptors or related proteins at the neuromuscular junction (NMJ). The NMJ, which has served as the classical model of the chemical synapse, uses acetylcholine as the neurotransmitter, and mutations of proteins involved in the signaling cascade lead to a variety of behavioral phenotypes. Mutants isolated after random chemical mutagenesis screening are summarized, and advances in the field resulting from these mutants are discussed.

The zebrafish is an animal model for biomedical research which has continuously increased in popularity over the past several decades. Its unique features make this small tropical fish an excellent model in a wide variety of biomedical fields. In addition to conventional whole-cell patch clamp, a plethora of genetic tools developed over the years, in combination with the transparency of the embryonic and larval stages, render zebrafish particularly amenable to optics-based physiological techniques, for example optogenetics [1] or calcium imaging [2, 3]. In this mini review we summarize advances in the study of synapses in zebrafish, focusing on the neuromuscular junctions (NMJs).

## Behavior mutants isolated by random mutagenesis

When random mutagenesis by use of *N*-ethyl-*N*-nitrosourea (ENU) was first applied to zebrafish in the 1990s [4], most of the mutants isolated by large-scale screening were identified on the basis of characteristic morphological abnormalities at the embryonic stage. In addition to these morphological mutants, a group of mutants with abnormal behavior was also isolated [5]. Mutants in this group were relatively slow to attract attention compared with the morphological or developmental phenotypes. Symptoms of these mutants ranged from total paralysis to reduced motility and circular swimming. Subsequent identification, one by one, of the genes underlying these behavioral phenotypes shed light on the neural system, furnishing new information on the function of the genes underpinning the motor system.

## Mutants of AChR subunits

**Fig. 1:**
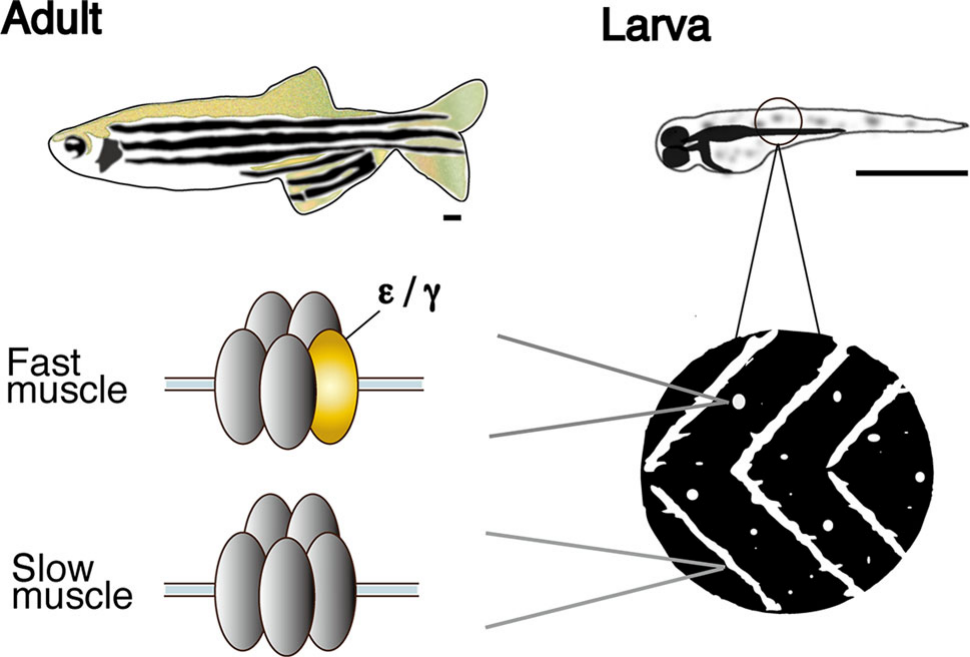
Schematic diagram of the zebrafish NMJ. In larvae, AChR clusters are located at the edge of slow muscle fibers and form chevron-shaped lines at the boundary of body segments, whereas those in fast muscle fibers are observed as round, punctate dots distant from the edges. AChRs in slow and fast fibers have subunits of different composition. *Scale* 1 mm

Many of the locomotion mutations mapped to genes coding for synaptic proteins in the NMJs. On the presynaptic side, synaptic vesicles containing acetylcholine are released from motor neuron endings in response to membrane excitation. Zebrafish mutants of pre-synaptic proteins, for example NSF [6] or the voltage-sensitive calcium channel [7], were identified and analyzed; these will not be discussed in this review. On the post-synaptic side, acetylcholine receptors (AChRs) receive acetylcholine released from the motor neuron endings and conduct inward currents through the open pore, leading to a cascade of events that culminates in the contraction of the muscle fiber. AChRs at NMJs comprise of five subunits (thus called pentamers): two α1 s, one β1, one δ, and one ε or γ, with the ε and γ subunits being interchangeable. The γ subunit is expressed early in development and, in mammals and fish, is replaced by the ε subunit as the animal matures [8, 9]. A notable exception to this long-held view, which will be discussed in detail below [10, 11] (Fig. 1), is the recent finding that NMJs in some types of zebrafish muscle fiber have pentamers that have neither the ε nor γ subunit but, instead, have two δ subunits.

**Fig. 2:**
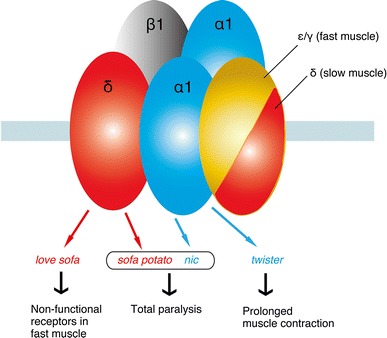
Zebrafish mutants of AChRs. Subunits harboring the mutation, their names, and the phenotypes are shown

Two null mutants were identified in zebrafish AChR subunits (Fig. 2). One mutant, *nic* [12], mapped to the α1 subunit and is a result of a splicing error that blocks the synthesis of the α1 subunit. Another mutant, *sofa potato* [13], mapped to the δ subunit. In contrast with *nic*, a full-length δ subunit is synthesized. However a conserved leucine residue is changed to proline near the N-terminus, which blocks the assembly of pentamers. In *nic* and *sofa potato*, AChR pentamers fail to form and the remaining normal subunits are retained in the endoplasmic reticulum [14]. These null mutants are completely paralyzed until their death approximately 7 days post-fertilization (dpf), but can be rescued by introducing a normal copy of the mutant subunit [13, 15]. The rescued null mutant can grow to adulthood and breed normally [15]. The subunit used for rescue can also be fused to a fluorescent molecule in the long intracellular loop, which makes the introduced protein visible.

The absence of a β1 mutant isolated from random mutagenesis screening is puzzling. The β1 subunit, a structural subunit which does not contribute to binding of the ligand, is absolutely necessary to form AChR pentamers [10, 16]. Therefore a null mutant of the β1 subunit will lack AChRs in the NMJ and, hence, detection by phenotype-based screening is expected to be straightforward. One possible explanation is duplication of the β1 gene in the zebrafish genome. In teleost genomes, some genes have duplicated copies resulting from fish-specific genome-wide duplication (FSGD) supposed to have occurred between 335 and 404 million years ago [17]. Two duplicated copies in zebrafish, β1a and β1b, encode subunits homologous to the mammalian β1. However, when expressed in Xenopus oocytes with other subunits, β1a did not lead to robust expression of functional receptors [10, 18]. Although it is possible the β1a subunit is functional in vivo, There are no convincing data showing that β1a can compensate for β1b when the latter is knocked out.

## Point mutations of AChR subunits cause striking phenotypes

A merit of random mutagenesis compared with reverse genetics is that random point mutations often lead to unexpected phenotypes and reveal previously unrecognized aspects of protein function. In zebrafish AChRs, the *twister* mutant [19] and the *love sofa* mutant [11] are such examples. The *twister* mutant has a point mutation in the α1 subunit whereas the *love sofa* mutant has a point mutation in the δ subunit (Fig. 2).

**Fig. 3:**
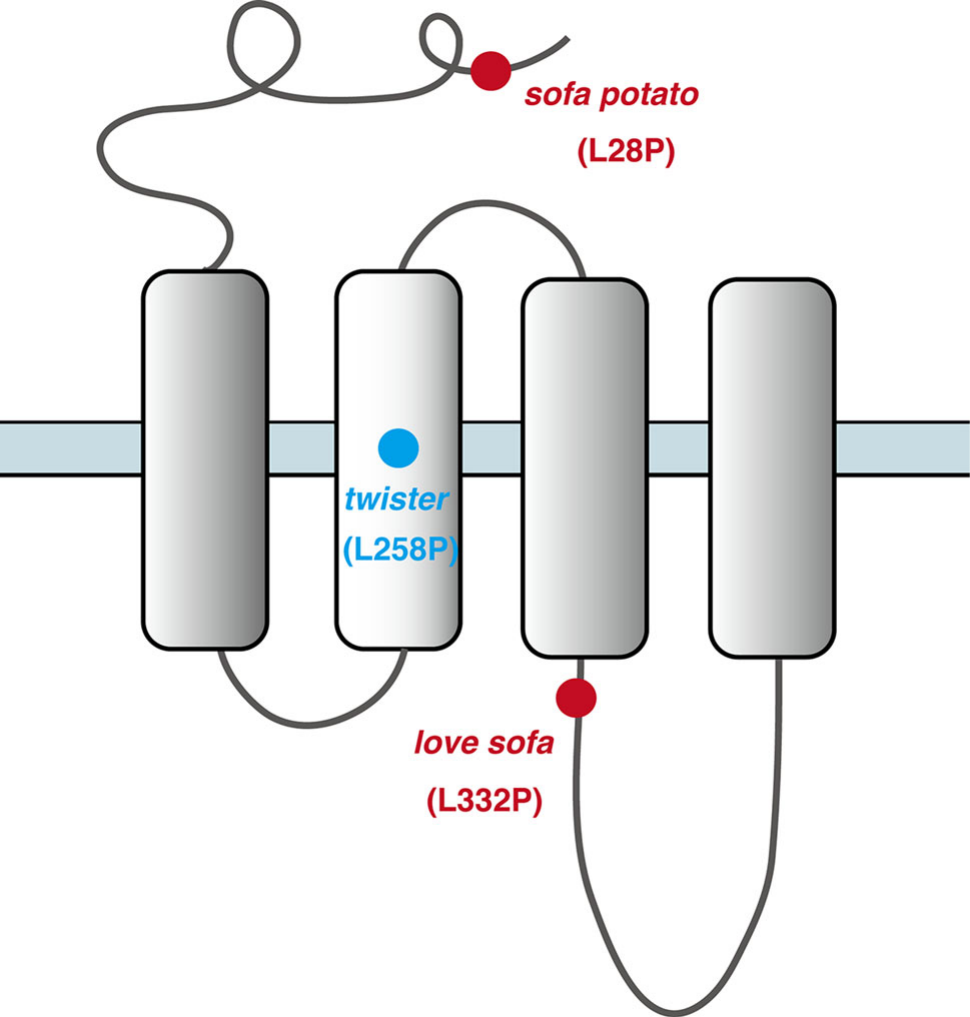
Locations of mutations in *twister*, *sofa potato*, and *love sofa* are indicated relative to the trans-membrane regions of the α (for *twister*) or δ (for *sofa potato* and *love sofa*) subunit. In each mutant, a leucine residue was changed to proline

The *twister* mutation mapped to a leucine residue (L258P) in the M2 trans-membrane region (Figs. 2, 3). AChRs containing the α1 subunit with the *twister* mutation have longer channel openings leading to a stronger, prolonged muscle contraction [20]. Fish homozygous for the *twister* mutation are embryonic lethal whereas heterozygotes for *twister* result in phenotypes linked with prolonged muscle contraction. Interestingly this phenotype can only be observed during early development [19], and heterozygotes recover from their behavioral defect as they age, which coincides with a change of synaptic current kinetics. This recovery is based on the developmental subunit switch from λ to ε [9].

The *love sofa* mutant, caused by a point mutation in the δ subunit, gives rise to a peculiar phenotype (Fig. 2). Similar to mammals, zebrafish skeletal muscle contains two fiber types, i.e. slow muscle fibers and fast muscle fibers (also called type I and type II fibers, respectively, in mammalian muscles) [21]. Fish homozygous for the *love sofa* mutation only form functional synapses in slow muscle fibers whereas fast muscle fibers have non-functional receptors that do not conduct currents in response to released acetylcholine. Although mutations in some muscle genes skew the proportion of fiber types as the skeletal system adapts to muscle degeneration [22], to the best of our knowledge, no mutation other than *love sofa* causes fiber type-specific phenotypes restricted to the synapse. This unexpected effect of the mutation arises from the different composition of subunits in the two types of fiber [11]. Slow muscle fibers lack ε/γ subunits and have two δs instead, which also underlies the different kinetics of AChRs [10] (Figs. 1, 2). This is, as stated above, a deviation from a traditional view of AChR subunit composition, in which ε or γ was believed to be a necessary component of AChRs at NMJs in vivo. The *love sofa* mutation is located in the basal region of the cytoplasmic loop between the third and fourth trans-membrane regions (Fig. 3), and is likely to affect the global structure of the AChR pentamer, rendering the subunit combination found in fast muscle fibers non-functional yet sparing that of the slow muscle fibers. These findings show that zebrafish is a useful model to investigate the difference between the NMJ of slow and fast muscle fibers at the molecular level.

## Rapsyn regulates AChR in a reciprocal manner

Rapsyn is a myristoylated cytoplasmic protein expressed in muscle cells that interacts directly with AChRs and is important in regulation of AChR clustering [23]. On the basis of a behavioral phenotype, a *rapsyn* mutant called *twitch once* has also been identified in zebrafish. This mutant has an escape response upon touch [24]. However the muscle contractions weaken and the swimming stops after a few tail bends. This phenotype is reminiscent of the muscle fatigue observed in human myasthenic patients and, interestingly, mutations in the human *rapsyn* gene cause the congenital myasthenic syndrome [25]. The *twitch once* mutant harbors a mutation in the tetratricopeptide repeat domain of the rapsyn protein and does not form AChR clusters at the synapse [24]. Instead AChRs are distributed diffusely on the plasma membrane.

Although rapsyn interacts with several proteins [26], its interaction with AChRs is best characterized and central to its function. Although rapsyn was originally identified as a factor that binds to AChRs at a 1:1 ratio, later analysis indicated that the stoichiometry is less rigid [27]. Analysis of the interaction of rapsyn with AChR subunits by use of conventional biochemical techniques proved onerous, and over the years several laboratories have used elegant techniques to unravel the mechanism of protein interaction [28, 29]. Notably, by use of chimeras of CD4 and AChR subunits, phosphorylation of the AChR β subunit was shown to intensify the binding of rapsyn to AChRs [30].

Rapsyn was initially believed to regulate AChR localization without any input from the AChR. More recent studies using the AChR null mutant (*sofa potato*) and cell-line systems revealed that the regulation is actually reciprocal [31, 32]. Rapsyn cannot localize to the synapse correctly in the absence of AChRs [13]. In particular, analysis of rapsyn in *sofa potato* revealed that rapsyn cannot reach the plasma membrane without AChRs and is retained in the Golgi apparatus [14]. When normal AChRs were introduced in *sofa potato*, the transport of rapsyn to the plasma membrane was restored. This effect of AChR on rapsyn was highly specific: the expression of a different AChR molecule, normally expressed in the brain [33], failed to restore rapsyn localization [14].

## Conclusion

Studies of mice and humans have revealed molecules involved in the development of post-synaptic structure at the NMJ, for example muscle-specific kinase (MuSK), dok-7, or Lrp4 [34–36]. Zebrafish mutants harboring mutations in these genes have also been identified. For example, a mutant of MuSK, *unplugged*, has defects in AChR distribution and in axon pathfinding [37, 38]. Further analysis of this MuSK zebrafish mutant revealed regulation by Wnt signals [39].

The zebrafish mutants discussed in this review provided valuable insights into the pathophysiology of some neurological disorders. *Sofa potato* and *twitch once* had mutations in genes later identified to cause human diseases [13, 24, 25, 40], and physiological study of *twister* indicated unappreciated benefits of subunit switch in myasthenic cases [9].

One notable feature of the zebrafish NMJ is its accessibility for optical, time-lapse studies in vivo. Ex-utero development combined with genetically introduced fluorescent molecules enable studies in which synapse formation can be observed in its entirety, both spatially and temporally [14, 41, 42]. Studies utilizing these unique approaches complement studies using human patients or mammalian animal models. Recent advances in genome-editing techniques make reverse genetics applicable to zebrafish [43]. Application of these new techniques will render the zebrafish system an even more useful model, which will continue to clarify the formation and function of synapses.
